# Hairless but no longer clueless: understanding glandular trichome development

**DOI:** 10.1093/jxb/erw339

**Published:** 2016-10-04

**Authors:** Johannes W. Stratmann, Carlton J. Bequette

**Affiliations:** Department of Biological Sciences, University of South Carolina, Columbia, SC 29208, USA

**Keywords:** Actin cytoskeleton, glandular trichome, *hairless* mutant, herbivory, SRA1, WAVE-regulatory complex.

**Glandular trichomes are remarkable biochemical factories that produce a wealth of secondary metabolites important for plant defenses against herbivores. However, little is known about their developmental biology. Studying the tomato *hairless* mutant, which develops distorted glandular trichomes and has compromised metabolic and defense capacity, Kang *et al.* (pages 5313–5324 in this issue) showed that the *SRA1* gene regulates not only trichome morphogenesis, but also aspects of secondary metabolism.**

Trichomes are hairlike structures found on many plant surfaces. Emerging from the epidermal cell layer, they develop into morphologically very diverse forms ranging from single cells to multicellular structures. Some are conspicuous – such as the multicellular trichomes in nettles or tomatoes – and cause a ‘hairy plant’ appearance. Some are smaller, but nevertheless obvious to people through essential oils they produce, such as the peltate trichomes of mint species (Lange and Turner, 2013); similarly, functions may appear obvious, as in the production of insect-trapping exudates by tobacco trichomes (Box 1). Other functions include regulation of transpiration, absorption of harmful UV-B radiation and plant defense (Wagner *et al.*, 2004; Vigneron *et al.*, 2005).

**Box 1. Trichomes in action**

Glandular trichome exudates trap fungus gnats (*Bradysia* spp.) with a similar efficiency to commercial ‘yellow sticky traps’. Sticky trichomes are known to reduce performance of small arthropod herbivores (Wheeler Jr and Krimmel, 2015).

**Figure:**
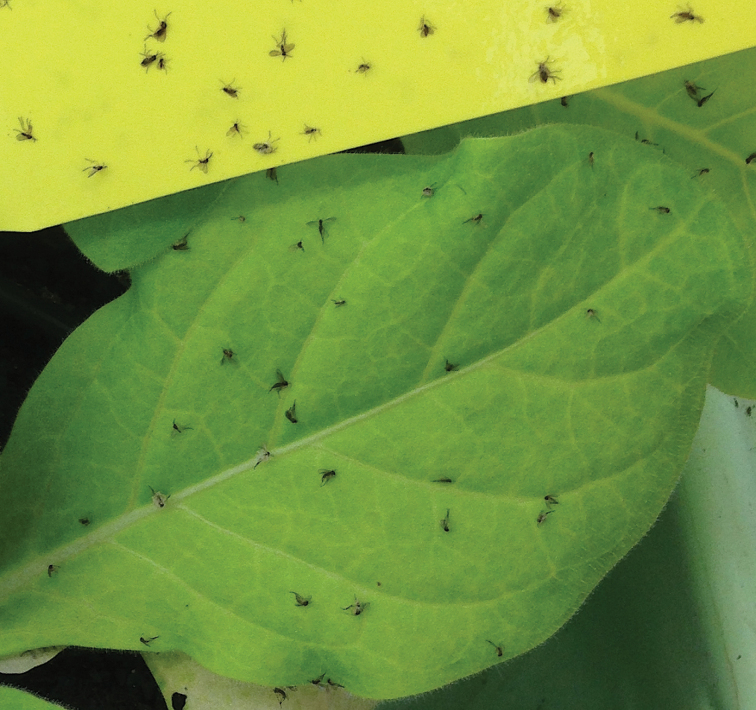

In contrast to non-glandular trichomes, glandular trichomes are metabolically highly diverse and synthesize, store and release secondary metabolites such as terpenoids, flavonoids and acyl sugars. Many of these chemicals are thought to function in defense against herbivores and pathogens, and they are also of great interest to human nutrition and medicine. To provide a compelling example, the alternative anti-malaria drug artemisinin, a sesquiterpene lactone, is produced in trichomes of *Artemisia annua* and its discovery by Youyou Tu resulted in her 2015 Nobel prize (Muangphrom *et al.*, 2016). With the availability of high-throughput metabolomic and genomic methods, much has been learned about the amazingly diverse secondary metabolism of glandular trichomes (Lange and Turner, 2013). They are clearly not simple hairs, but highly evolved plant organs.

We have an advanced understanding of the development of trichomes in Arabidopsis. However, it possesses only single-celled, non-glandular trichomes. Tomato provides many advantages pertinent to research on glandular trichomes. It not only has several different types, but also offers a sequenced genome, a detailed genetic map, routine genetic transformation, high diversity within the tomato clade, and availability of a number of trichome mutants.

Through the work of Gregg Howe’s group, we already know some basic facts about glandular trichome development in tomato, such as the involvement of *JAI-1* (*COI1*), coding for the receptor for the plant hormone jasmonic acid (Li *et al.*, 2004*a*), *CHALCONE ISOMERASE 1* (*CHI1*) (Kang *et al.*, 2014), and the so far unidentified *ODORLESS-2* gene (Kang *et al.*, 2010*a*). Mutants in these genes all exhibit a distorted trichome phenotype, compromised synthesis of terpenoids and flavonoids, and reduced resistance against herbivores. Characterization of these mutants represents a promising start for delineating glandular trichome development in tomato, but until now they had not offered mechanistic insights into the regulation of development at the molecular level.

## First glimpse into glandular trichome development at the molecular level

The paper by Kang *et al.* (2016) is the culmination of previous efforts aimed at characterizing the *hl* mutant and represents a significant breakthrough in understanding the development of multicellular glandular trichomes. The name of the mutant is misleading: *hl* mutants do have glandular trichomes (Reeves, 1977), but an earlier study by Kang *et al.* (Kang *et al.*, 2010*b*) showed that they are smaller and distorted (see Box 2): only the density of types I and VI on leaves is reduced, and type VI exhibits reduced levels of sesquiterpenes and flavonoids. This altered defense metabolite profile correlates with a reduced resistance to herbivory. Kang *et al.* (2016) now demonstrate by map-based cloning that the *hl* mutant harbors a mutation in the *SRA1* (*SPECIFICALLY RAC1-ASSOCIATED*) gene. When the *hl* mutant was transformed with the full-length wild-type tomato *SRA1* sequence, the wild-type phenotype was fully reconstituted.

**Box 2. The *hairless* mutant**

The *hairless* mutant harbors a mutation in the *SRA1* gene, a component of the pentameric WAVE regulatory complex (Sra1 and blue boxes). SRA1 (also known as PIROGI) interacts with NAP1 (NCK-associated protein) and WAVE (also known as SCAR) via the C-terminal VCA domain of WAVE. WAVE also interacts with NAP1 via ABI, and with BRICK1/HSPC300. The complex is activated via small GTPases (G) such as RAC1 or ROP2, which target SRA1, and in turn regulates actin assembly by the ARP2/3 (actin-related protein 2/3) complex (Chen *et al.*, 2010; Seaman *et al.*, 2013; Yanagisawa *et al.*, 2013). A dysfunctional WRC in *hl* results in distorted trichomes, reduced synthesis of sesquiterpenes and flavonoids, reduced resistance to herbivores and brittleness of the stem. Nomenclature shown is according to Chen *et al.* (2010).

**Figure:**
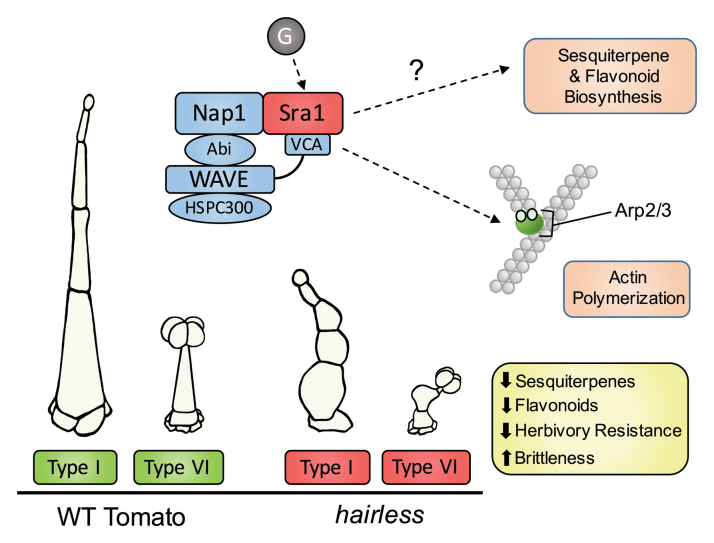

*SRA1* is conserved among multicellular organisms and is one of five components that constitute the WAVE regulatory complex (WRC) (Box 2). The WRC regulates actin assembly by the ARP2/3 complex (Seaman *et al.*, 2013; Yanagisawa *et al.*, 2013). Actin filaments play an important role in cell development, such as enabling the targeted delivery of cell wall building blocks to the extracellular space (Yanagisawa *et al.*, 2013).

This finding is significant because it is the first report to demonstrate a role for the WRC in the development of glandular trichomes. Moreover, the WRC connects the actin-cytoskeleton network with secondary metabolism and plant defense against herbivores. It provides a lever for more in-depth studies of the molecular mechanisms underlying glandular trichome development. SRA1 and the WRC also regulate trichome development in Arabidopsis (Yanagisawa *et al.*, 2013), although there are fundamental differences between non-glandular and glandular trichomes.

The other novel outcome of this work is that the WRC affects the synthesis of a select group of secondary metabolites, mainly some sesquiterpenes and flavonoids. Currently it is unknown what the role of the actin network is in these processes. Furthermore, the *hl* mutant, which is impaired in the synthesis of certain sesquiterpenes and flavonoids, is less resistant against tobacco hornworm caterpillars (*Manduca sexta*), although it has wild-type levels of monoterpenes, glycoalkaloids, acylsugars and antinutritional proteins. This indicates that defense against herbivores is a concerted action of diverse groups of defense compounds, and that each group represents an essential component for overall resistance.

## New avenues for glandular trichome research

A dysfunctional actin-cytoskeletal network is expected to result in more severe developmental defects in other plant organs. Indeed, tomato *sra1*/*hairless* mutants have brittle stems and Kang *et al.* (2016) attribute that to improper cell wall development, which also requires a functional actin cytoskeleton. However, beyond that, *hl* mutants look fairly normal (Kang *et al.*, 2010*b*). In contrast, the Arabidopsis *sra1* null mutant exhibits much more severe phenotypes, with changes ranging from development to reproduction (Li *et al.*, 2004*b*). It cannot be excluded that this is due to the nature of the *sra1* mutation in *hl*, possibly generating a C-terminally truncated SRA1 protein. The truncated protein may still carry out other functions, but lack a domain or motif specific to trichome development. Using CRISPR/Cas9 or RNAi technology, *sra1* null mutants could be generated, and the *hairless* mutant then transformed with truncated versions of SRA1 to define functional domains, motifs, or critical amino acids. It will probably be challenging to unravel detailed molecular dynamics of the WRC in tomato, but straightforward deletion/complementation studies will go a long way towards defining functions of the five WRC constituents. By linking this to glandular trichome phenotypes, it should be possible to learn how they develop – for example, how cell division and cell shape formation in multicellular glandular trichomes is regulated. This would be aided by inclusion of additional tomato trichome mutants.

The other genes that deserve more attention are those making up the ARP2/3 complex. It would be interesting to find out whether tomato plants mutated or silenced for these genes display an altered secondary metabolite profile. This would directly link actin organization and secondary metabolism.

Not much is known about the activation of the WRC by developmental signals. SRA1 plays a central role within the WRC by interacting not only with small GTPases, which mediate activating signals emanating from the plasma membrane, but also with the VCA domain of WAVE, which directly activates the ARP2/3 complex and thus actin polymerization (Box 2). The VCA domain is inactive when bound to SRA1. Activation of SRA1 by a GTPase results in release of VCA, which then activates ARP2/3. Phosphorylation of certain WAVE residues may also contribute to the activation of the WRC (Chen *et al.*, 2010; Mendoza *et al.*, 2011; Yanagisawa *et al.*, 2013). Since the WRC is important for plant defenses (Kang *et al.*, 2010*b*; Kang *et al.*, 2016), and the defense hormone jasmonic acid also affects trichome development and secondary metabolism (Li *et al.*, 2004*a*), there is an interesting link that could be further investigated.

## Future biofactories?

Kang *et al.* (2016) have demonstrated that tomato SRA1 is part of the WRC and is a key regulatory factor for glandular trichome development and secondary metabolism. We know this is important in terms of understanding function, but as our knowledge develops it may also have wider implications – as already noted, the secondary metabolites produced by trichomes have a range of practical applications. Particularly, advanced insights into WRC function could inspire breeding efforts to utilize plants as biofactories that produce desirable metabolites in their glandular trichomes.
